# A Synopsis of Sardinian Studies: Why Is it Important to Work on Island Orchids?

**DOI:** 10.3390/plants9070853

**Published:** 2020-07-06

**Authors:** Michele Lussu, Michela Marignani, Roberta Lai, Maria Cecilia Loi, Annalena Cogoni, Pierluigi Cortis

**Affiliations:** 1 Dipartimento di Scienze della Vita e dell’Ambiente, Università degli Studi di Cagliari, 09130 Cagliari, Italy; marignani@unica.it (M.M.); robi.lai@gmail.com (R.L.); loimc@unica.it (M.C.L.); cogoni@unica.it (A.C.); pierluigi.cortis@gmail.com (P.C.); 2 Istituto Regionale per la Floricoltura (IRF), 18038 Sanremo, Italy

**Keywords:** continental island, evolution, geographic isolation, Mediterranean Basin, Orchidaceae, Sardinia

## Abstract

Biological and ecological investigations of islands are crucial to explain ecosystem functioning. Many studies on island biodiversity are carried out on oceanic islands. In contrast, information on continental islands, such as those in the Mediterranean Sea, is very often fragmented in space and time. Here, a synopsis of the Orchidaceae of Sardinia is presented based on literature surveys and recent botanical field studies. Our final list comprises of 64 species and 14 genera: thirteen species and subspecies were recognized as endemic and four new species were recorded for the flora of the island: *Anacamptis palustris* (Jacq.) R.M. Bateman, Pridgeon & M.W. Chase; *Himantoglossum hircinum* (L.) Spreng; *Orchis italica* Poir.; and *Platanthera kuenkelei* subsp. *kuenkelei* var. *sardoa* R.Lorenz, Akhalk., H.Baumann, Cortis, Cogoni & Scrugli. This orchid richness reflects the geological history of the island that was linked to the mainland several times, facing long periods of isolation. We also discuss a critical point-of-view of the biodiversity shortfalls still problematic for insular orchids. Indeed, within the Mediterranean Basin, the greatest amount of endemism occurs mainly on large islands, and, despite a long history of botanical exploration in European countries, many of them are scarcely investigated. This annotated synopsis shows the potential of continental islands to understand trends in ecology and evolution. Further studies are required to complete our knowledge of the orchid diversity on continental islands in order to propose scientific-based conservation programs to preserve these unique taxa.

## 1. Introduction

Islands are natural laboratories to develop and test evolutionary and ecological theories [1,2,3,4,5]. Geographic isolation is one of the main causes of speciation [1]. When populations of the same breeding group are separated, they face independent evolutionary histories defined by natural selection, genetic drift, adaptation and colonization to local conditions [1,6]. Driven mainly by allopatry [7], island biodiversity is influenced by the distance from the mainland, the size and the age of the island [8]. Nevertheless, the idea that islands should not be seen merely as target areas for plant colonization but also as “halting places” was proposed by Darwin [9] but surprisingly has only recently been demonstrated [10,11]. On the basis of their origin, islands might be divided into two groups: oceanic islands, which have a volcanic origin and do not lay on the continental shelves, and continental islands, which lay on the continental shelves and have been linked with the mainland at some point in their past. Species–area and species–elevation relationships are the most common patterns adopted in ecology as descriptors of species richness because their increase corresponds to greater environmental diversity [12]. The relation between species recorded and area observed, species–area relationships (SARs), is considered as one of ecology’s few laws [12] and it is widely used to explain several ecological and biogeographic theories as the equilibrium theory of island biogeography [3]. The elevational diversity gradient (EDG) is influenced by temperature, precipitation and productivity which drastically change along elevational gradient shaping niches diversity [13]. Then, elevation is considered to be a surrogate of topographic complexity and habitat diversity [8,14,15,16,17]. Studies on insular biodiversity have been historically conducted on oceanic islands [3,4,5,7] and only recently on continental islands [8]. With more than 12,000 islands, the Mediterranean Basin is a hotspot of biodiversity [18,19]. Many of these islands are continental and originated during the Holocene, but few, such as Stromboli, Vulcano, Ischia, Pantelleria and Santorini, are volcanic. Each island or islet represents a small universe where ecological and evolutionary patterns have led to a unique ecosystem [19]. However, due to its complexity, the total number of plant species in the Mediterranean Basin is still uncertain and varies, depending on the author, from 25,000 to 30,000 [20,21]. The current structure, distribution and species diversification of Mediterranean flora have been deeply affected by dramatic palaeogeographic events and climatic changes occurred during the late Tertiary and Quaternary [22,23,24]. Within Angiosperms, the cosmopolitan family of Orchidaceae is one of the most species-rich and new species are described every year [25]. The extraordinary variability of their flowers and the peculiarity of their relationships with pollinators has attracted humans from remote times, allowing them to be adopted as model in biological studies [9,26]. Nevertheless, the concept of species in orchids still generates a vibrant debate and the literature about which character is taxonomically relevant is particularly rich [26,27,28]. Sardinia is the second largest island in the Mediterranean Basin and has a remarkable plant diversity [29]. One of our aims is to fill and summarize the distributional information (Wallacean shortfall [30]) on orchid across the island, which is a pivotal feature required by different international protection tools such as IUCN Red List and Important Plant Areas (IPAs) [31] and European ones (e.g., European Red List of Vascular Plants [32]), as uncertain knowledge on species biogeography easily leads to an inadequate prioritization [33,34,35,36]. Circa 85% of the island surface still lacks a detailed biogeographic study [37] and the field effort is concentrated mainly on the coasts and mountains [38]. This richness originated mainly as a portion of the continental biota transported during the fragmentation processes of the continental platform. Indeed, during the Early Oligocene, Sardinia, Corsica and the Balearic Islands were part of a mountain chain present in Iberia [37] that migrated to the actual location in the Western Mediterranean experiencing episodes of reunification with the continental platforms during the Messinian salinity crisis and Pleistocene glaciations [31,32,39]. A further route of plant colonization is long-distance dispersal (LDD). In the Mediterranean Basin, this mechanism has been proposed to explain the colonization of *Quercus* suber from Africa to Southern Europe, as well as the colonization of species with dust-like seeds, such as *Platanthera kuenkelei* subsp. *kuenkelei* var. *sardoa* [40,41]. The most important published contribution on orchids of Sardinia dates back to 1990 [42], followed, eighteen years later, by a PhD thesis [43], which represents the most updated available synthesis for the island, including 60 species in 15 genera. Since studies on Mediterranean plant biogeography are often run at species levels but rarely higher levels [8] and due to the recent progresses in different biological fields, we decided to update knowledge on Sardinian orchids. The aims of this paper are: (i) to update the check-list of Sardinian Orchidaceae presenting some notes about the observed orchid taxa; (ii) to propose an identification key; (iii) to relate insular orchid species and endemisms richness with area and maximum elevation; and (iv) to discuss the relevance of orchids in island conditions for evolutionary and conservation research and why they should have more attention.

## 2. Results

### 2.1. Current Knowledge

Our final list comprises 64 species and 14 genera: thirteen species are recognized as endemic (Table 1, Table 2 and Table 3). The chorologic spectrum of the endemic units is dominated by Sardo-Corsican taxa (6), followed by Sardinian (5), Sardo-Sicilian (1) and Sardo-Tunisian (1) ones (Table 3). Flowering period begins in January and ends in November (Table 4). The identification key is proposed in Supplementary File 1.

### 2.2. Local Conservation Status

Out of 64 species, 33 were classified as Least Concern, 18 as Vulnerable, 8 as Endangered and 5 as Critically Endangered. In contrast with the IUCN Red List of Threatened Species, in our analysis, six species were classified as Vulnerable from Least Concern (*Anacamptis laxiflora, A. pyramidalis, Cephalanthera damasonium, C. rubra, Epipactis palustris* and *Neotinea tridentata*), three from Near Threatened to Vulnerable (*Limodorum trabutianum, Platanthera algeriensis* and *Serapias nurrica*), four from Least Concern to Endangered (*Himantoglossum hircinum, Orchis brancifortii, O.italica* and *O. purpurea*), two from Least Concern to Critically Endangered (*Anacamptis palustris* and *Epipactis helleborine* subsp. *muelleri*) and one from Near Threatened to Critically Endangered (*Dactylorhiza elata* subsp. *sesquipedalis*).

### 2.3. Species–Area and Species–Elevation Relationships

**Figure 1:**
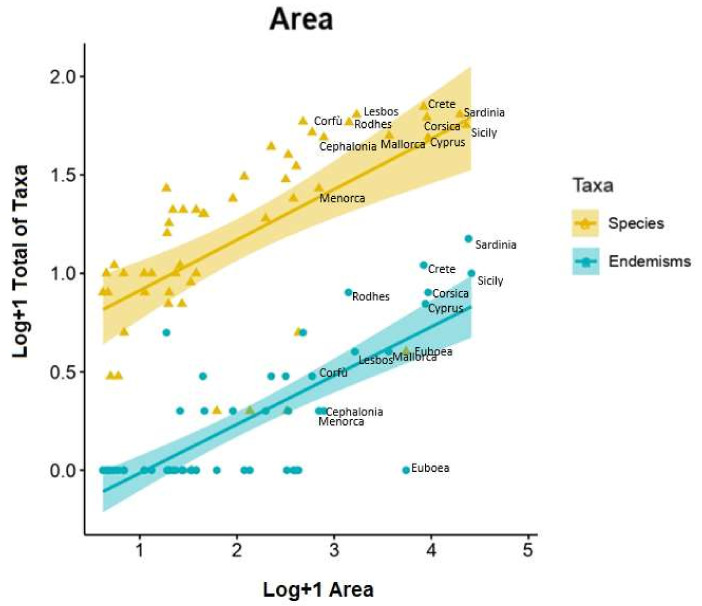
Endemic and species area-relationships of orchid richness for Mediterranean islands. For a better graphic representation, only islands with area greater than 500 km^2^ are named.

The results of the regression on species richness indicated the two predictors explained 34.4% of the variance (R2 = 0.3171, F1,50 = 21.58, *p* < 0.01). It was found that species richness was significantly predicted by area (*β* = 0.37, *p* =0.00836) but not by elevation (*p* = 0.07293). Our results on endemisms show that area and elevation explained 56.9% of the variance (R2 = 0.3171, F2,49 = 32.28, *p* < 0.01); the endemisms richness of an island was significantly predicted by area (*β* = 0.739, *p* = 1.93e-07) but not by elevation (*p* = 0.8585) (Figure 1).

### 2.4. Key Shortfalls

In the last decades, the number of orchid species recorded in Sardinia has varied from 55 in 1990 to 64 in this review, reflecting the increasing efforts of previous and current researchers to fill gaps in knowledge (Supplementary Files 2 and 3). We are still far from an exhaustive knowledge on Sardinian orchids and several gaps still affect our knowledge about them. The main causes probably are because ecological and evolutionary studies are hindered by gaps about identity, evolution, distribution and ecological dynamics of biodiversity [44]. At the core of all the shortfalls nests the notion of species [45]. The classification of organisms into different groups (species) is still a crucial debate. Indeed, it interferes with all other aspects of biodiversity, and thus the scarcity of information about the number of species occurring is one of the most relevant deficiencies in biodiversity [45]. Since orchids are highly specialized organisms where eco-ethological adaptations are not always reflected in genotypes, species circumscription has rarely found agreement among taxonomists [26,46,47]. Thus, this debate is a losing game in the presence of a disproportion of knowledge between morphofunctional data and molecular data, as we recorded for many Sardinian orchids (see Table 1 and Supplementary Files 2 and 3). Indeed, molecular data useful for phylogenetic reconstruction, Darwinian shortfall [48], are a crucial tool to understand evolutionary patterns in Mediterranean orchids [49]. From our field experience, we noticed the diversity of endemic entities and we strongly believe that a lumper approach could lead to the loss of some or all the nuances that orchids evolve in insular conditions. Indeed, only two (*Ophrys holoserica* subsp. *chestermanii* and *O. normanii)* out of five endemic taxa exclusive of Sardinia have been investigated from a phylogenetic perspective, underlining the relevance of this analysis to clarify species boundaries [50]. The remaining three, *O. subfusca* subsp. *liveranii*, *O. fusca* subsp. *ortuabis* and *O. panattensis,* are still not investigated and their phylogenetic positions is only partially supported [51]. In our case, the scarcity of the island’s surface investigated [37] raises several questions about the distribution of species already recorded as well as for those that have a potential distribution area on the island. Thus far, one of the major problems we encountered is that many of the previous publications are based on species distribution and rarely on their abundance. If Marignani et al. (2014) [52] asked if time is on our side or not, the local recent discovery and similarly rapid disappearance of *Anacamptis palustris*, and the current status of *Ophrys scolopax* subsp. *apiformis* and *Dactylorhiza elata* subsp. *sesquipedalis,* in line with the global reports [36], do not allow to waste precious time. In the last years, there has been an increase of studies based on functional traits in relation to reproductive success rather than their taxonomic or phylogenetic affiliations [53,54], the Raunkiæran shortfall [55], these studies must be conducted in parallel between island and continental species to understand, for example, the success of some taxonomic groups. Ecological studies on functional traits in Sardinian orchids have been focused mainly on pollination biology of endemisms [56,57,58]. However, pollinators are still not clear for some of them such as *Ophrys scolopax* subsp. *conradiae* and *Platanthera kuenkelei* subsp. *kuenkelei* var. *sardoa*, implying a partial circumscription of these species. Nevertheless, to make our data more usable and contextualized in a context that goes beyond the borders of the island, we responded to TRY’s initiative to contribute to new datasets for TRY version 6 with our unpublished data on functional traits (Lussu et al. unpublished). Given the current knowledge and supported by technical tools such as bioinformatic software and less expensive molecular analyses, the integration of results from different area of natural sciences have the potential to fill gaps in shorter times. This, we know, is a race against time because biodiversity is disappearing as *Anacamptis palustris* and *Dactylorhiza elata* subsp. *sesquipedalis* have taught us. Due to the lack of knowledge described above, our final list should not be considered definitive. Instead, it should be used to plan further studies on orchids in insular conditions.

## 3. Materials and Methods

### 3.1. Study Area

#### 3.1.1. Topography

Sardinia is located in the central western Mediterranean Sea between 38°51′ and 41°15′ latitude N and 8°80′ and 9°50′ E longitude with an area of 24,090 km², being 270 km long and 145 km wide. The nearest island is Corsica, 11 km way, and the Italian peninsula is 187 km away.

Relevant mountain peaks are: Punta La Marmora (1834 m) and Monte Limbara (1362 m). The main wide alluvial valleys and flatlands are the Campidano and the Nurra. Seasonal rivers are relevant, while perennial rivers include the Tirso, 151 km, the Coghinas, 115 km, and the Flumendosa, 127 km. The only natural lake is Lago di Baratz, all the others are artificial. Large, shallow, salt-water lagoons are characteristic of the coast, particularly relevant is Molentargius lagoon in the city of Cagliari used for centuries for salt production.

#### 3.1.2. Geology

The age of schistore sedimentary rocks varies from Lower Cambrian to Lower Carboniferous. These metamorphites result intruded by pleitonic granitoid rocks of Permo-Carboniferous age, belonging to the wide Sardinian-Corsican batholith [59]. Thick marine carbonatic sediments lie discordant on the Paleozoic Basement eroded during the Permo-Triassic period. A very thick Cenozoic succession constituted by continental and marine sediments, marles and limestones, of Eocene to Pliocene ages, rests on the Mesozoic sequence or, more frequently, directly on the Paleozoic Basement. Acid to basic volcanites of Oligocene–Miocene and Pliocene–Pleistocene volcanic cycles are associated to the above-mentioned sediments. Finally, detritic, prevalently continental, deposits of the ancient and recent Quaternary discontinuously cover all the previous geological formations [59].

#### 3.1.3. Climate

Sardinia has a Mediterranean climate with two macrobioclimates: Mediterranean pluviseasonal oceanic and temperate oceanic. The continentality varies from weak semihyperoceanic to weak subcontinental including four classes, while eight thermotypic horizons ranging from lower thermomediterranean to upper supratemperate and seven ombrothermic horizons from lower dry to lower hyperhumid are described. Annual temperature ranges from 11 to 17 °C. Precipitations are typical of autumn and winter months and they vary from 400 to 1100 mm per year; spring and summer are usually hot and dry. Snowfalls are generally rare, but quite frequent in the highest mountain chains. The most relevant winds are the cold Mistral and the hot Scirocco from Sahara [60].

#### 3.1.4. Plant Diversity

Sardinia, as a part of Mediterranean Basin, is classified within one of the 34 most important biodiversity hotspot in the world [19]: there are 2333 species, of which 347 are endemics [61,62]. The dominant chorological element is the Stenomediterranean (29%), followed by the Eurasian (17%) and the Eurimediterranean (16%) [63].

#### 3.1.5. Human Impact

It is still unclear when *Homo sapiens* colonized Sardinia; however, first evidence of a stable human presence is recorded during the Upper Paleolithic [62,63]. During the Bronze Age, the Nuragic civilization arose. Through the following centuries, coasts were colonized in succession from Phoenicians, Romans, Vandals, Goths, Byzantines and Saracens, leaving the inner part of the island particularly isolated, causing, therefore, a strong genetic isolation in Sardinians.

The most ancient and relevant human activity on the island is sheep grazing [64]. In the last fifty years, the island has suffered of land abandonment and an increasing human pressure on coasts caused mainly by new touristic settlements. A constant and inadequate landscape management is a direct cause of soil salinification and desertification [65]. Since 1956, Sardinia hosts four North Atlantic Treaty Organization (NATO) military bases in strategic locations for a total of 213.6 sq km.

Three regional parks have been established, namely Regional natural park of Porto Conte (1999), Regional Natural park of Molentargius-Saline (1999) and Regional Natural park of Tepilora, Sant’Anna and Rio Posada (2014). and recognized as a biosphere reserve by UNESCO in 2017. In addition, three National parks were created: the archipelago of La Maddalena National Park in 1994, Asinara National Park in 1997 and Gulf of Orosei and Gennargentu National Park in 1998. National and Regional Parks cover an area of 1141 km^2^

### 3.2. Check-List of Sardinian Orchids

We carried out a literature search through ISI^®^ Web of Science, Scopus and Google Scholar as well as through cross-referencing. The initial search terms for the query (23 February 2020) included “Orchids” AND “Sardinia”; we also performed a second query including each taxon recorded in Scrugli [42] and Lai [43] AND “Sardinia”. In addition, we implemented our investigation in the NIH genetic sequence database (GenBank) and the global archive of plant traits [66] using the previous keywords. We included studies meeting the following criteria: (i) performed in Sardinia; (ii) involved taxa listed in Sardinia; and (iii) published in peer-reviewed journals. Because of its useful local relevance, we also took into account the grey literature not published in peer-reviewed journals or published in Italian, German and French. To assess the conservation status of each taxa, we consulted IUCN Red List of Threatened Species databases, and we also investigated their legal protection. A specific search was conducted on the check-lists of Europe and Italy [42,43,62,67,68,69,70,71]. We decided to base our list on the work of Lai [43], updating it with new records and nomenclature changes. In addition to the bibliographic research, we also included unpublished data collected during almost 10 years of fieldwork carried out across the island during the decade (2009–2018). We included flowering period data taken from literature and integrated with our field observations. Descriptions of species and hybrids were prepared from both living specimens and herbarium material. New samples were deposited at the Herbarium CAG of Università degli Studi di Cagliari. Species identification was carried out adopting Lai [43] (2008) and [72] Kühn, Pedersen and Cribb (2019). A separate search was conducted on the orchid flora of the other Mediterranean Islands (Supplementary File 4). Nomenclature follows The International Plant Name Index.

### 3.3. Assessment of Local Conservation Status

To assess the local conservation status of orchid species, we used the terminology and the set of criteria and adopted by IUCN Red List of Threatened Species because it is widely recognized. However, Despite the data required by IUCN, our assessment is a simplified version based on the number of populations and the number of mature plants. Following Liu et al. (2005) [72], our categories consist of: Least Concern, >5000 individuals and many locations; Vulnerable, >5000 mature individuals and only 1–5 locations; Endangered, >250 mature individuals; Critically Endangered. <50 mature individuals or only known locations threatened with destruction; and Data Deficient, data were not sufficient.

### 3.4. Species–Area and Species–Elevation Relationships

Multiple regression analysis was used to test if the area and elevation significantly predicted species and endemic richness in Mediterranean Basin. We enumerated the orchid flora of 52 islands in the Mediterranean biogeographic region according to Médail and Quézel [18] and Thompson [23]. species–area relationships (SARs), endemics–area relationships (EARs) [19,29,73] and species–elevation relationships were investigated using standard linear regression. For SARs and EARs, the log–log representation of the power model was applied [12,74]. Statistical analyses were performed using R Core Team 2015.

## 4. Discussion

Both Wallace [75] and Darwin [1] underlined the role of islands to explain biological processes and their relevance in biogeography. In studies of island systems, particular emphasis has been placed on how biogeographic processes and island traits influence patterns of species diversity (e.g., [4,5,76,77]). The high level of biodiversity recorded in the Mediterranean shows the potential of continental islands to explain bio-ecological patterns [23]. Despite ecological investigations such as species–area or species–elevation relationships are ordinarily tested on oceanic island contest [5,11], studies on continental archipelagos are becoming relevant, also due to the availability in grey literature of long-term data [78,79,80]. The larger is the island, the more ecological niches available are expected and island area is a predictor of orchid species richness in the Mediterranean Basin. Big islands, such as Sardinia, are definitely more diversified in terms of habitats exploitable from drought tolerant genera such as *Ophrys* L. and *Anacamptis* Rich., which represent a significant portion of the Mediterranean orchid flora. On the contrary, elevation does not play a dominant role in orchid species richness and only six islands exceed the elevation of 1500 m. This might depend on several climatic oscillations encountered by this region during the Quaternary, which led to the current subtropical and xeric climate prompting the radiation of bee orchids. Indeed, simultaneously, the same oscillations would have led to extinction of the mesophilic taxa or pushing them to refuge in the very few niches survived on peaks [24]. Paleoclimatic species distribution models have shown that the Sardo-Corsican plate were probably the refuge of *Dactylorhiza* species during the Last Glacial Maximum (LGM) [81]. Indeed, allotetraploid taxa such as those of the *majalis* complex (ssp. *lapponica*, ssp. *majalis*, ssp. *traunsteinerii*) originated well before the last ice age. This would explain the limited distribution of *D. elata* subsp. *sesquipedalis* in Sardinia in one area, the Ogliastra, considered a glacial refugium from Médail et al. (2009) [24]. *D. elata* subsp. *sesquipedalis* prospers exclusively in nitrogen-poor substrates and it has a very narrow distribution similar to other refugia of *D. majalis* s.l. [82,83]. As reported for other plant families [84], within the Mediterranean Basin orchid endemic taxa show ecological difference compared to their congeners in the mainland. In *Cyclamen*, narrow endemic taxa occur in different habitats to their widespread species [84]. We observed the same pattern in at least three *Ophrys.* While *O. fusca* subsp. *ortuabis* clearly grows in association with *Salvia rosmarinus* Spenn., *O. holoserica* subsp. *chestermanii* and *O. normanii* prefer shady positions and deep soil if compared to their analogous mainland species. We also noticed that these three taxa exhibit specific phenotypic differences with their counterparts. Despite the large number of zoological studies, the Foster’s rule is still underestimated as well as controversial in plant sciences [85]. This ecogeographic rule states that on islands species tend to be bigger (insular gigantism) or smaller (insular dwarfism) due to resources availability [86]. Given the massive distributional and morphological data on Mediterranean orchids and the numerous cases of Mediterranean vertebrates that support this theory [68,70,87], orchids would be a consistent model to test in plants.

When compared to the other two big islands in West and Central Mediterranean, i.e., Corsica and Sicily, the uniqueness of the Sardinian orchid flora lies in its isolation and its habitat diversity. Indeed, despite the short distance that separates Sardinia and Corsica (only 11 km), their orchid flora diverges considerably because Corsican substrates are formed by a granite backbone while Sardinian are more heterogeneous. Specifically, Sardinia has fewer orchid genera but a higher number of species that prefer calcareous soils. Thus, Corsica hosts typically Apennine genera such as *Epipogium* Borkh. and *Gymnadenia* R.Br., suggesting its strong linkage with the continental plate. Moreover, all Corsican orchid endemisms have a Sardo-Corsican chorology while Sardinia hosts several single island endemisms, emphasizing the importance of Sardinia in driving speciation processes within the Mediterranean Basin. When compared with Sicily, instead, Sardinia has an analogous orchid flora but Sardinia displays the presence of the monospecific genus *Gennaria* Parl. and a higher number of endemisms as a consequence of its isolation. Notably, Sicily is only 3 km from the Italian Peninsula, and, therefore, its orchid flora is particularly influenced by this proximity, as suggested by the greater species richness of the genus *Dactylorhiza* Neck. ex Nevski and *Epipactis* temperate-boreal genera. In comparison with East Mediterranean islands, Sardinia is species poorer, but it is richer in terms of single island endemisms. The explanation may lie, again, in the remoteness of Sardinia in space and time that might have influenced processes of founder events or immigration by LDD generally accepted to explain distributional pattern in *Dactylorhiza* [81]. Adriatic, Aegean and Ionian Archipelagos are made up of dozens of islands and islets closely interconnected with each other and with the mainland. Moreover, their proximity to the continental plates, their recent geological separation and strong winds blowing from the mainland may have reduced genetic isolation on each island, providing seed dispersion within archipelagos. 

Although DNA-based analyses have been particularly useful in resolving phylogenies of the main Mediterranean clades, by far intragenetic mechanisms of some charismatic Mediterranean orchid genera are still a challenge for present and future research [49]. In Sardinia, haplotype network constructions have revealed the complex phylogeographic mechanisms of the local genetic diversity in *Epipactis* Zinn and *Platanthera* Rich. [41,88]. In the same way, the recent use of Next-Generation Sequencing and its integration with morphometric, distributional and reproductive biology datasets has contributed to the knowledge about evolutionary mechanisms within the major groups of aggregate species such as the *fusca* group in *Ophrys* and the *helleborine* group in *Epipactis*. For instance, Bateman et al. (2020) [49] attempted to construct the phylogeny of the genus *Ophrys* noting that *fusca* branch obtained by the cladistic tree and RAD-seq tree is definitely long, suggesting a high rate of evolution. In Sardinia, this group is encompassed by four geographically, morphologically and ethologically well-defined microspecies. An interesting evolutionary question would be to analyze more precisely molecular patterns in co-occurring taxa at insular level [89,90]. Indeed, unfortunately, no molecular investigation involving these microspecies on a single island has yet been conducted, contributing to a lack of taxonomic clarity and therefore a proliferation of names not yet tested with scientific rigor. A locally defined sampling might be useful to define species and then be applicable in other insular contexts. 

There are many eco-evolutionary questions that continental islands raise: Do orchids on continental and volcanic islands follow the same evolutionary patterns? At what stage of their evolutionary life are endemisms on the islands? What is the most suitable concept of species to describe these organisms that are still not very differentiated or poorly studied? What are the most suitable tools to protect orchids in this contest? We are confident that one of the most relevant threats to island orchids in the Mediterranean is the lack of knowledge on several fundamental aspects of their life such as pollination, their ecology and their genetic linkage. Irregular knowledge on species and their distribution has deep consequences on their conservation [44]. The variation of the number of orchid species recorded in Sardinia over time depends on the evolution of the concept of species from 1990 to today, but it also reflects the increase of a research based on extensive campaigns. However, this situation is not exclusive to Sardinia only, and we believe it can be extended to many other Mediterranean contexts. For instance, the only available data on the status of *Dactylorhiza elata* subsp. *sesquipedalis* concern exclusively the very small Italian population and no other information are available for other countries where it is recorded, i.e., France, Spain and Portugal [91]. We should also reflect more on the potential of non-academic involvement to gain data useful to science. Indeed, the integration of non-orthodox data sources into research has led to interesting phenological and distributional results [92,93]. In Sardinia, the discovery of new species would not have been possible without the field support of enthusiasts and orchid lovers. Furthermore, studies on endemic taxa have contributed to clarify strategies of evolution within the Orchidaceae family such as pollinator convergence [28,50,57] or define mechanisms of colonization in a complex system such as the Mediterranean Basin [41]. The ambiguous morphology of *Ophrys normanii* was the reason it was identified for decades as the hybrid between *O. holoserica* subsp. *chestermanii* and *O. tenthredinifera* and named *O. × maremmae* nsubsp. *normanii* (J.J. Wood) H. Baumann & Künkele. AFLP analysis, GC-EAD analyses and pollination experiments clarified that *O. normanii* is phylogenetically isolated by its putative parents and the sharing of *Bombus vestalis* as pollinator with *O. holoserica* subsp. *chestermanii* depends on convergent evolution [57]. On the other hand, Pavarese et al. (2011) [41] pointed out the role of LDD in defining the current Sardinian flora. In fact, they found that the single population of *Platanthera kuenkelei* subsp. *kuenkelei* var. *sardoa* is more closely related with Algerian samples than with *Plantanthera bifolia* (L.) Rich. from the Italian Peninsula. Linking the African, Arabian and Eurasian plates, the Mediterranean Basin is represented by groups of islands with intricate geopolitical history and this leads to a heterogeneous biodiversity management with direct consequence on conservation [52,91,92,93]. Nevertheless, the need of further research to fill gaps in knowledge has been already expressed [26,94]. 

Although much work has been done, much more effort is required to fill the gaps in knowledge with the non-secondary purpose of preserving biodiversity from anthropic pressure. Climate changes towards overheating affect species in quantity and distribution [95] and Ongaro and collaborators [96] suggested that several species will increase their range. The nine species investigated by Ongaro et al. [96] are overspread all over the island and they tolerate a wide range of habitats. Consequently, it is speculative to make predictions about the future distribution of taxa with restricted geographic location not knowing or knowing only partially what explains their actual occurrence. In Europe, where the human pressure lasted for centuries, orchids are dramatically threatened by habitat reduction and fragmentation [97,98], limiting orchid lifespan [99,100,101] and affecting their reproductive success. Nevertheless, habitat management might positively affect local orchid richness: for example, mowing could preserve orchids because it allows higher light intensities [102]. Even in Sardinia, mowing management actions could explain the orchid richness on roadside. In fact, in April and May, to prevent summer fires, local municipalities promote a haphazard mowing of grasses, but this time span corresponds to the blooming season of many orchid species. A better timing in the scheduled mowing, taking into account the life cycle of plant species—not only orchids—would certainly be significant to conserve plant diversity. Human impact might also influence plants affecting pollinator communities [103,104]. Globally, the main causes of the decrease of insects are landscape management and intense agricultural activities [98,105,106]. These pressures are generally not intense in low populated islands such as Sardinia [66] but, unfortunately, studies on pollinators distribution are absent in Sardinia. In Mediterranean islands, anthropic pressure is directly expressed with touristic coastal settlements whose impact is particularly consistent. It is not a case that the two most endangered species in Sardinia, *Anacamptis palustris* and *Dactylorhiza elata* subsp. *sesquipedalis* are located on the coast in fragile habitats such as humid herb grasslands (Habitat Directive (92/43/EEC)). Their peculiar life cycle exposes orchids to a greater danger of extinction when compared to other plants [107,108]. The Habitat Directive (92/43/EEC) is an important European tool to protect biodiversity but it does not preserve orchids directly. Indeed, it mentions “semi-natural dry grasslands and scrubland facies on calcareous substrates (Festuco-Brometalia) (important orchid sites), code 6210”. Currently, Italy does not have a more exhaustive national regulation to directly protect orchids, and thus it has delegated the legislative provisions to the regions and autonomous provinces. The Autonomous Region of Sardinia has not issued any law yet. Although at regional level the requests for a regional law for the protection of the flora have not yet been accepted, positive local conservation actions are guided by small municipalities that have shown, despite their economic constraints, particular interests in developing protection and conservation initiatives. Under this light, at the Mediterranean level, the answers to questions of which conservation approach would be more adequate or which species or groups of species should have a conservation priority are really intricate to provide. A necessary step to deal with is to really understand the potential integrated and coordinated studies between islands and, at least countries members of the European Community, to make valid decisions before time begins to run against us. Many studies adopt the island of Sardinia to develop new approaches on conservation program both at theoretical and practical levels ([109,110,111] and references therein); nonetheless, a deeper analysis on orchids is required because of their peculiar biology. Indeed, we should remember that, on the 2018 IUCN Red List of Threatened Species, four of the five extinct orchids are island endemism and this should be an alarm bell to warn us of the risk island orchid biodiversity currently faces.

## 5. Conclusions

Thus far, this synopsis is the most comprehensive analysis of studies on orchids on Sardinia and it could be helpful to reflect on how much we know about orchid diversity in continental island conditions: due to the complexity of the biology and evolution of orchids, we are aware that this list will face, in time, some thrilling changes. Knowledge about orchids is still deeply affected by critical gaps. Defining this lack is essential to draw future investigations on these charismatic organisms because of the peculiar location, geological, history and biogeographic gradient of Sardinia. Moreover, the exclusive and often fragile ecosystems of islands stimulate the rise of new questions, the development and testing of new theories at every ecosystem’s level and the investigation on how species interact. Hence, especially for islands where organisms have followed unique evolutionary trends, more integrated studies are necessary to fill existing knowledge deficits, in order to answer new eco-biological questions and to develop adequate programs to protect these intriguing lifeforms.

## Figures and Tables

**Table 1:** Species list and databases review. In TRY Plant Trait Database, columns refer to: ObsNum, Number of Observations; ObsGRNum, number of geo-referenced observations; ObsPubNum, number of public observations; MeasNum, number of measurements; MeasGRNum, number of geo-referenced measurements; TraitNum, number of traits.

	IUCN Red List		GenBank Items	TRY Plant Trait Database				
Species	Global	Local		ObsNum	ObsGRNum	MeasNum	MeasGRNum	TraitNum
*Anacamptis*								
*A. collina*	Least Concern	Least Concern	6					
*A. fragrans*	Least Concern	Least Concern	2					
*A. laxiflora*	Least Concern	Vulnerable	70					
*A. longicornu*		Least Concern	38					
*A. palustris*	Least Concern	Critically Endangered	24					
*A. papilionacea* var. *papilionacea*	Least Concern	Least Concern	4					
*A. papilionacea* var. *grandiflora*		Least Concern	4					
*A. pyramidalis*	Least Concern	Vulnerable	25	244	31	447	87	120
*Cephalanthera*								
*C. damasonium*	Least Concern	Vulnerable	86					
*C. longifolia*	Least Concern	Least Concern	106	329	57	827	334	111
*C. rubra*	Least Concern	Vulnerable	13	1	469	4	113	
*Dactylorhiza*								
*D. elata* subsp. *sesquipedalis*	Near Threatened	Critically Endangered						
*D. insularis*		Vulnerable	2					
*Epipactis*								
*E. gracilis*		Vulnerable						
*E. helleborine*		Least Concern	1734	509	117	1458	738	145
*E. helleborine* subsp. *muelleri*	Least Concern	Critically Endangered	13	74		164		74
*E. microphylla*		Least Concern	41	100	12	236	8	81
*E. palustris*	Least Concern	Vulnerable	162	271	25	427	25	120
*E. tremolsii*		Vulnerable	1					
*Gennaria*								
*G. diphylla*	Least Concern	Least Concern	11					
*Himantoglossum*								
*H. robertianum*	Least Concern	Least Concern	41					
*H. hircinum*	Least Concern	Endangered						
*Limodorum*								
*L. abortivum*	Least Concern	Least Concern	97	93		234		
*L. trabutianum*	Near Threatened	Vulnerable	1					
*Neotinea*								
*N. lactea*	Least Concern	Least Concern	7					
*N. maculata*	Least Concern	Least Concern	16					
*N. tridentata*	Least Concern	Vulnerable	9					
*Neottia*								
*N. nidus-avis*	Least Concern	Least Concern	84	434	21	701	23	127
*N. ovata*		Vulnerable	34					
*Ophrys*			84					
*O. apifera*	Least Concern	Least Concern	102					
*O. bombyliflora*	Least Concern	Least Concern	16	1				
*O. eleonorae*		Least Concern						
*O. exaltata* subsp. *morisii*		Least Concern	9					
*O. funerea*		Endangered	5					
*O. fusca*	Least Concern	Least Concern	79					
*O. fusca* subsp. *ortuabis*		Endangered	0					
*O. garganica*		Vulnerable	64					
*O. holoserica* subsp. *annae*		Vulnerable	0					
*O. holoserica* subsp. *chestermanii*		Least Concern	0					
*O. incubacea*		Least Concern	17					
*O. lutea*	Least Concern	Least Concern	26					
*O. normanii*	Endangered	Endangered	0					
*O. panattensis*		Endangered	0					
*Ophrys scolopax subsp. apiformis*		Critically Endangered	1					
*O. scolopax.* subsp. *conradiae*		Vulnerable						
*O. sicula*		Least Concern	11					
*O. speculum*	Least Concern	Least Concern	18					
*O. sphegodes* subsp. *praecox*		Vulnerable	0					
*O. subfusca* subsp. *liveranii*	Least Concern	Least Concern	0					
*O. tenthredinifera*	Least Concern	Least Concern						
*Orchis*								
*O. anthropophora*	Least Concern	Least Concern	61					
*O. brancifortii*	Least Concern	Endangered	2					
*O.italica*	Least Concern	Endangered						
*Orchis mascula* subsp. *ichnusae*		Vulnerable	0					
*O. provincialis*	Least Concern	Least Concern	26	1				
*O. purpurea*	Least Concern	Endangered	29					
*Platanthera*								
*P. algeriensis*	Near Threatened	Vulnerable	4					
*P.kuenkelei* subsp. *kuenkelei* var. *sardoa*	Critically Endangered	Critically Endangered	8					
*Serapias*								
*S. cordigera*	Least Concern	Least Concern	12	2		3		2
*S. lingua*	Least Concern	Least Concern	19	6		17		9
*S. nurrica*	Near Threatened	Vulnerable	5					
*S. parviflora*	Least Concern	Least Concern	11	3		19		12
*Spiranthes*								
*S. aestivalis*	Data Deficient	Vulnerable	12					
*S. spiralis*	Least Concern	Least Concern	47					

**Table 2:** Orchid hybrids recorded in Sardinia.

Hybrid	Parental Species	Distribution
*Anacamptis × bornemannii* Asch.	*Anacamptis papilionacea × Anacamptis longicornu*	W-Medit.
*Anacamptis × caccabaria* Verguin	*Anacamptis laxiflora × Anacamptis papilionacea*	Medit.-Atl.
*Anacamptis × sarcidani* Scrugli et Grasso	*Anacamptis laxiflora × Anacamptis longicornu*	Endem. SA
*Ophrys × barbaricina* M. Allard et M.P.Grasso	*Ophrys speculum × Ophrys morisii*	Endem. SA
*Ophrys × cosana* H. Baumann et Kunkele	*Ophrys bombyliflora × Ophrys incubacea*	W-Medit.
*Ophrys × daissiorum* (H. Baumann, Giotta, Künkele, Lorenz & Piccitto) P. Delforge	*Ophrys holoserica* subsp. *chestermanii × Ophrys morisii*	Endem. SA
*Ophrys × domus-maria* M.P. Grasso	*Ophrys apifera × Ophrys morisii*	Endem. SA
*Ophrys × fernandii* Rolfe	*Ophrys bombyliflora × Ophrys speculum*	W-Medit.
*Ophrys × heraultii* G. Keller ex Schrenk	*Ophrys tenthredinifera× Ophrys speculum*	Medit.
*Ophrys × laconensis* Scrugli et Grasso	*Ophrys exaltata subsp. morisii × Ophrys tenthredinifera*	Endem. SA
*Ophrys × maladroxiensis* Scrugli, Todde e Cogoni	*Ophrys exaltata subsp. morisii × Ophrys holoserica* subsp. *annae*	Endem. SA
*Ophrys × manfredoniae* O. & E. Danesch	*Ophrys incubacea × Ophrys tenthredinifera*	W-Medit.
*Ophrys × sommieri* E.G. Camus ex Cortesi	*Ophrys bombyliflora × Ophrys tenthredinifera*	Medit.
*Ophrys × spanui* P. Delforge	*Ophrys holoserica* subsp. *annae × Ophrys tenthredinifera*	Endem. SA-CO
*Ophrys × sulcitana* Scrugli, Todde e Cogoni	*Ophrys holoserica* subsp. *annae × Ophrys bombyliflora*	Endem. SA
*Ophrys × tavignanensis* H. & J.M. Mathé & M. Pena	*Ophrys eleonorae × Ophrys incubacea*	W-Medit.
*Orchis × penzigiana* A. Camus nsubsp. *sardoa* Scrugli et Grasso	*Orchis provincialis × Orchis mascula subsp. ichnusae*	Endem. SA
*Serapias × ambigua* Rouy	*Serapias cordigera × Serapias lingua*	Medit.-Atl.
*Serapias × cortoghianae*, Grasso M.P	*Serapias nurrica × Serapias cordigera*	W-Medit.
*Serapias × semilingua* E.G. Camus et al.	*Serapias lingua × Serapias parviflora*	Medit.-Atl.

**Table 3:** List of endemic taxa and their distribution.

Chorological Rank	Taxa
Endem. SA	*Ophrys holoserica* subsp. *chestermanii*
Endem. SA	*Ophrys normanii*
Endem. SA	*Ophrys fusca subsp. ortuabis*
Endem. SA	*Ophrys panattensis*
Endem. SA	*Ophrys subfusca* subsp. *liveranii*
Endem. SA-CO	*Ophrys holoserica* subsp. *annae*
Endem. SA-CO	*Ophrys funerea*
Endem. SA-CO	*Ophrys exaltata subsp. morisii*
Endem. SA-CO	*Ophrys scolopax* subsp. *conradiae*
Endem. SA-CO	*Ophrys sphegodes* subsp. *praecox*
Endem. SA-CO	*Orchis mascula* subsp. *ichnusae*
Endem. SA-SI	*Orchis brancifortii*
Endem. SA-TU	*Platanthera kuenkelei* subsp. *kuenkelei* var. *sardoa*

**Table 4:** Flowering period of Sardinian orchids during the year.

	I		II		III		IV		V		VI		VII		VIII		IX		X		XI		XII	
*Anacamptis*																								
*A. collina*																								
*A. fragrans*																								
*A. laxiflora*																								
*A. longicornu*																								
*A. palustris*																								
*A. papilionacea* subsp. *grandiflora*																								
*A. papilionacea* subsp. *papilionacea*																								
*A. pyramidalis*																								
*Cephalanthera*																								
*C. damasonium*																								
*C. longifolia*																								
*C. rubra*																								
*Dactylorhiza*																								
*D. elata* subsp. *sesquipedalis*																								
*D. insularis*																								
*Epipactis*																								
*E. helleborine*																								
*E. helleborine* subsp. *muelleri*																								
*E. microphylla*																								
*E. palustris*																								
*E. persica* subsp. *exilis*																								
*E. tremolsii*																								
*Gennaria*																								
*G. diphylla*																								
*Himantoglossum*																								
*H. robertianum*																								
*H. hircinum*																								
*Limodorum*																								
*L. abortivum*																								
*L. trabutianum*																								
*Neotinea*																								
*N. lactea*																								
*N. maculata*																								
*N. tridentata*																								
*Neottia*																								
*N. nidus-avis*																								
*N. ovata*																								
*Ophrys*																								
*O. apifera*																								
*O. bombyliflora*																								
*O. eleonorae*																								
*O. exaltata* subsp. *morisii*																								
*O. funerea*																								
*O. fusca*																								
*O. fusca* subsp. *ortuabis*																								
*O. garganica*																								
*O. holoserica* subsp. *annae*																								
*O. holoserica* subsp. *chestermanii*																								
*O. incubacea*																								
*O. lutea*																								
*O. normanii*																								
*O. panattensis*																								
*O.scolopax* subsp. *apiformis*																								
*O. scolopax.* subsp. *conradiae*																								
*O. sicula*																								
*O. speculum*																								
*O. sphegodes* subsp. *praecox*																								
*O. subfusca* subsp. *liveranii*																								
*O. tenthredinifera*																								
*Orchis*																								
*O. anthropophora*																								
*O. brancifortii*																								
*O. italica*																								
*O. mascula* subsp. *ichnusae*																								
*O. provincialis*																								
*O. purpurea*																								
*Platanthera*																								
*P. algeriensis*																								
*P.kuenkelei* subsp. *kuenkelei var. sardoa*																								
*Serapias*																								
*S. cordigera*																								
*S. lingua*																								
*S. nurrica*																								
*S. parviflora*																								
*Spiranthes*																								
*S. aestivalis*																								
*S. spiralis*																								

## Supplementary Materials

The following are available online at https://www.mdpi.com/2223-7747/9/7/853/s1, Supplementary File 1: Identification key to the orchids of Sardinia; Supplementary File 2: scientific literature concerned Sardinian orchids; Table Supplementary File 3: grey literature on Sardinian orchids; Supplementary File 4: literature on distribution of Mediterranean orchids.
